# Identifying social prescribing core outcomes using a Delphi approach: findings and future directions

**DOI:** 10.24095/hpcdp.46.1.02

**Published:** 2026-01

**Authors:** Maureen C. Ashe, Anna M. Chudyk, Margaret Lin, Gurkirat Singh Nijjar, W. Ben Mortenson, Theresa Pauly, Robert Petrella, Kathy L. Rush, Bobbi Symes, Sian Tsuei, Kate Mulligan

**Affiliations:** 1 Department of Family Practice, University of British Columbia, Vancouver, British Columbia, Canada; 2 College of Pharmacy, University of Manitoba, Winnipeg, Manitoba, Canada; 3 Home and Community Care Services, Fraser Health Authority, Surrey, British Columbia, Canada; 4 Department of Occupational Science and Occupational Therapy, University of British Columbia, Vancouver, British Columbia, Canada; 5 GF Strong Rehabilitation Research Program, Vancouver, British Columbia, Canada; 6 International Collaboration on Repair Discoveries, Vancouver, British Columbia, Canada; 7 Department of Gerontology, Simon Fraser University, Vancouver, British Columbia, Canada; 8 Centre for Studies in Family Medicine, Schulich School of Medicine and Dentistry, Western University, London, Ontario, Canada; 9 School of Nursing, University of British Columbia, Okanagan Campus, Kelowna, British Columbia, Canada; 10 United Way British Columbia, Burnaby, British Columbia, Canada; 11 Faculty of Health Sciences, Simon Fraser University, Burnaby, British Columbia, Canada; 12 Dalla Lana School of Public Health, University of Toronto, Toronto, Ontario, Canada

**Keywords:** determinants of health, outcomes research, public health, seniors

## Abstract

**Introduction:**

Although social prescribing is a growing global health and social movement, no Delphi studies have determined which outcomes are critical to assess. Our aim was to identify a core outcome set based on feedback from diverse user groups of people who could be affected by (e.g. adults ≥ 60 years) or who can affect (e.g. providers, researchers) social prescribing.

**Methods:**

Following standard guidelines for Delphi studies, we developed a two-round online survey with a focus on Canadian perspectives. We asked participants to rate 21outcomes as “critical” (7–9 on a 9-point scale), “important but not critical” (4–6 points) or “not important” (1–3 points). We provide a subgroup description of findings from older adult/family and friend perspectives.

**Results:**

Round 1 was completed by 74 people from 10 user groups and Round 2 by 52people from eight user groups (70% retention). Ratings between rounds were generally consistent. Seven outcomes met the “critical” threshold. No outcomes were excluded. Critical outcomes focused on mental health, physical and social functioning, and well-being. Participants commented on environmental (e.g. resources, care delivery) and equity factors.

**Conclusion:**

This study identified seven critical outcomes to consider in evaluations of social prescribing research and interventions. Future investigations should investigate how contextual and personal factors might influence outcomes and identify specific instruments (e.g. questionnaires, performance-based tests) to assess each outcome. Identification of outcomes is a continuous process, requiring regular updates as results may change due the ongoing evolution of social prescribing and other factors.

**Highlights**

- Social prescribing is an emerging health and social care model, but a list of outcomes that are the most important to assess has not yet been published.
- Having a core outcome set would help maintain consistency between studies for subsequent synthesis and to support practice.
- We conducted a Delphi study with different user groups, including adults 60 years and older and their families or friends, to determine which outcomes were critical.
- After the second of two survey rounds, seven outcomes that focused on mental health, physical and social functioning, and well-being were selected as critical.
- This is the first iteration of a social prescribing core outcome set; this set may change over time and depend on where it is applied.

## Introduction

The social prescribing movement is a health and social model of care that is growing globally.^1^,^2^ Social prescribing is described as a means of addressing people’s unmet social needs by connecting them with resources.^3^ Social prescribing can take a number of approaches, from a light touch that lets people know about community resources (“signposting”) to a holistic social prescribing hub with a team-based approach that can include “prescriptions” to community-based activities and resources that address health-related social needs and involve link workers who support people as they engage with these resources.^4^

Although the available evidence indicates that social prescribing shows promise, previous research has been limited by small studies,^4^ a lack of rigorous study designs^5^ and incomplete information on program implementation.^4^ Inconsistencies in the outcome measures used in studies of social prescribing impede the comparison of data across these studies.^6^ A lack of standardized outcomes might also affect program implementation, making it difficult to evaluate, improve and sustain implemented programs.^7^

Having a core outcome set could improve the consistency of study designs and facilitate meta-analyses.^8^ We were unable to identify any applicable core outcome sets that had been published despite that the most recent definition (based on the results of a Delphi study with experts in social prescribing from 26 countries) stated the need to use outcomes to evaluate the effects on the individual.^3^ Examples of impacts included “nonmedical, health-related social needs, health and well-being (physical, mental, social), satisfaction, clinical and nonclinical supports and services (e.g. demand, costs) and the community.”^3^^,p.8^ Well-being was the most frequently cited outcome in studies with adults 18 years and older.^9^

Although social prescribing programs are available for all ages,^1^ our focus is people as they age and, in particular, older adults—a large and growing demographic.^10^ Our previous systematic reviews^4^,^9^,^11^ focused on adults 40 years and older. Social prescribing may be especially important for older adults in the wake of reports of loneliness and social isolation during the pandemic.^12^

Recognizing the importance of engaging people with lived experience in the development of the core outcome sets,^13^ we were especially interested in the perspectives of older people and their families. Moreover, people who are directly affected need to be involved in developing solutions to challenges in order to bring about change^14^ in, for example, health and social care. It is also possible that excluding the recipients of an intervention such as social prescribing from a Delphi study could result in missing important outcomes.^13^ Further, including multiple perspectives may help identify those outcomes that are best tailored to the needs of recipients of social prescribing interventions, the programs and the community. 

The aim of this study was to identify a core outcome set for use in future social prescribing research trials with older adults, based on feedback from multiple diverse user groups. 

## Methods

***Ethics approval***

This study received ethics approval from the Behavioural Research Ethics Board at The University of British Columbia (H22-03569). We published our protocol^6^ and registered it with the Core Outcome Measures in Effectiveness Trials (COMET) database (https://www.comet-initiative.org/Studies/Details/2364). 

***Delphi study***

The aim of this prospective Delphi study^15^-^20^ was to identify critical outcomes for use in social prescribing research trials with adults aged 60 years and older. We followed guidance from *The COMET Handbook*^8^,^21^ to help identify and choose suitable measures for research trials to create “an agreed standardized collection of outcomes, known as a core outcome set … which should be measured and reported in all trials for a specific clinical area.”^22^^,p.1^

We conducted two rounds of an online Delphi survey to rate 21 outcomes for use in social prescribing research trials. These outcomes were grouped into domains based on a published taxonomy^23^ and our 2024 modified umbrella review.^9^

We delivered the 21-item list of outcomes to participants using DelphiManager (COMET Initiative, Liverpool, UK) in both rounds of the Delphi survey.

***Participants***

We invited people from diverse user groups—people who could affect or are affected by social prescribing^24^—to identify critical core outcomes to use in social prescribing. We focused our recruitment efforts within Canada to support national research and practice, but as social prescribing is relatively new to Canada, with the first evaluations commencing in 2018 in Ontario,^25^ we also identified potential participants from, for example, the United Kingdom. In British Columbia, where many of our research team are located, social prescribing began as a demonstration project in early 2020 in approximately 20 sites across the province, and the program continues to expand.^26^

Although the optimal number of Delphi panel members has not been established, it has been suggested that 30 to 50 people may be appropriate for a group with similar perspectives.^27^ We therefore tried to recruit an even larger number of participants in order to gather a variety of viewpoints. Diverse perspectives among panellists may also contribute to the coproduction process^28^ especially for interventions that focus on well-being,^29^ equity^30^ and sustainability and that take into account the “community paradigm,” which argues that “public services should work with the insight of people and communities to be effective and sustainable.”^31^^,p.1^

***Recruitment***

We extended invitations to individuals in the “social prescribing” user groups identified in our published protocol^6^ and based on our 2013 framework for older adults’ mobility in the community.^32^ These user groups included researchers (who could “test” social prescribing), health care providers (who could make referrals), community groups and social service providers (nonprofit or volunteer groups that could deliver social prescribing interventions), trainees (including students and fellows who could provide feedback and ensure the sustainability of social prescribing interventions), link workers (“community connectors” or “navigators”), data and implementation scientists, and ethicists as well as managers, decision-makers and policy-makers. 

We searched online for potential participants using terms such as “social prescribing” and keywords related to each user group and location (initially within Canada, and then wherever social prescribing was conducted), and identified publicly available email addresses. An author [MCA] sent email invitations to at least 10 (if possible) potential participants from each of the user groups, to a total of 131 email invitations (Table 1).

**Table 1:** Number of individual emails sent to recruit participants to pre-identified social prescribing user groups

Group number	User group	Recruitment emails sent, n
1	Link workers	22
2	Researchers	20
3	Trainees^b^	16
4	Health care providers	15
5	Community groups^c^ or social service providers	12
6	Data scientists	10
7	Ethicists	10
8	Implementation scientists	10
9	Policy-makers, managers, decision-makers	10
10	Family caregiver groups	6
**Total **		**131**

^a^ Identified in Esfandiari et al.^6^ and based on Schiller et al.^32^

^b^ Including students and fellows.

^c^ Including nonprofit and volunteer groups. 

We recruited older adults (≥60 years) and their family members or friends because of the need to understand their perspectives,^13^ in keeping with the coproduction approach in social prescribing.^1^ To recruit individuals, we worked with a British Columbia–based research service to post our ethics-approved recruitment materials online. Participants aged between 60 and 110 years were eligible to take part. We also posted ethics-approved recruitment material on our research laboratory’s social media account. 

We did not provide a stipend to study participants.

***Delphi survey rounds***

Round 1 of the Delphi survey started on 22 May 2024 and closed on 8 July 2024 (7 weeks). Round 2 started on 25 September 2024 and closed on 7 November 2024 (6 weeks). 

In our introductory emails to participants, we provided information on the study and access to DelphiManager, and assigned each participant a unique study number. We deliberately limited the materials sent to participants to prevent unintentional bias, providing them with the consent form (which included some background information) and instructions on how to participate in each round. 

We asked participants to rate each of the 21 outcomes on a nine-point scale from one (“not important”) to nine (“critical”). We categorized the responses into three groups: critical outcomes (7–9 points); important but not critical outcomes (4–6 points); and not important outcomes (1–3 points). Participants could also say that they were “unable to score” a response or to leave the question unanswered. 

In Round 1, participants could also suggest and rate additional outcomes to include in the next survey round. Only the person who suggested an additional outcome was able to rate it. Two researchers [MCA, AC] reviewed and adjudicated the suggested outcomes. 

Between rounds, participants were provided with a summary of their responses and results from their user group. 

***Delphi research team***

Core members of the Delphi research team were from the following user groups: community groups, decision-makers, health care providers, researchers and trainees. The study lead for recruitment and data analysis [MCA] is a professor and physiotherapy researcher who practised in the community setting and is an older adult. All team members were invited to complete the surveys; the study lead [MCA] did not provide ratings.

***Analysis***

We used SPSS version 30.0 (IBM, Chicago, IL, US) and Excel (Microsoft Corp., Redmond, WA, US) to clean and descriptively analyze data. We used the Excel Crosstabs function to identify how participants rated outcomes, using the category labels “critical,” “important but not critical” and “not important.” We did not compare participants’ ratings between rounds (e.g. to assess the stability of responses) as we only conducted surveys at two time points.^27^ We also did not to ask participants or research team members to rank outcomes because previous research has found results to be similar when outcomes are rated versus ranked, but the process of ranking is more difficult.^33^ We analyzed the ratings given by participants who self-identified in the older adults and family or friends/caregivers group separately from those of the other user groups, which we analyzed collectively.

***Definition of consensus used in this study***

We defined consensus in advance of this study. An outcome was included when at least 75% of participants rated it as critical (7–9 points) with less than 20% rating it as not important (1–3 points). Conversely, we excluded an outcome if more than 75% of participants deemed it not important with less than 20% of participants rating it as critical.^6^

## Results

**Figure 1:**
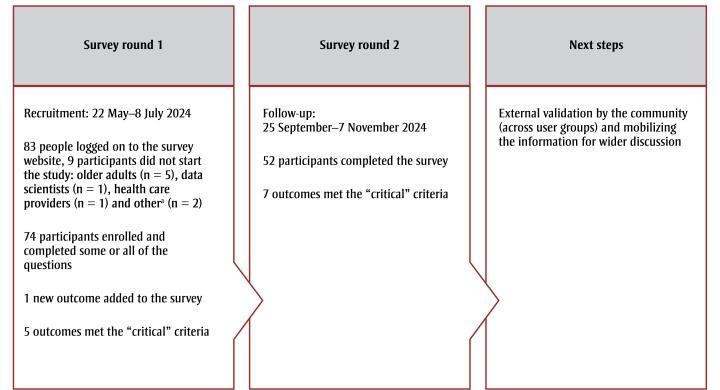
Flow diagram of study methods

^a^ No additional information is available. 

Between 22 May 2024 and 8 July 2024 (7weeks), 83 participants logged on to the study website. Nine participants did not start the survey: older adults (n=5); other (no additional information is available) (n=2); data scientists (n=1); and health care providers (n=1) (Figure 1).

No data scientists or ethicists in Canada or elsewhere enrolled in the study or chose to use either of these user group designations, despite the invitations emailed to potential participants (n=20). It is also possible that social support providers (or any participant) identified themselves as belonging to a different user group.

A total of 73 participants completed and one person partly completed Round 1. These 74 participants represented 10 user groups, including the nine social prescribing user groups recruited via email plus the group of older adults and their families or friends recruited via the research platform (Table 2). In Round 1, 59 (80%) of the participants were from Canada, 9 (12%) from the United Kingdom and 6 (8%) from Australia, Ireland or the United States. When asked if they had delivered or received social prescribing, 52 (70%) participants said no, 19 (26%) said yes and three (4%) did not respond.

**Table 2:** Number and proportion of participants in user groups at Delphi survey Rounds 1 and 2

User group	n (%)	
	Round 1	Round 2
Older adults and family or friends/caregivers^a^	24 (32)	14 (27)
Researchers and implementation scientists^a^	19 (25)	14 (27)
Community groups^b^ or social service providers	10 (14)	5 (10)
Trainees^c^	8 (11)	7 (13)
Health care providers	6 (8)	5 (10)
Link workers	3 (4)	3 (6)
Other^d^	2 (3)	2 (4)
Policy-makers, managers, decision-makers	2 (3)	2 (4)
Total	74	52

**Note: **Each participant self-selected their user group.

^a^ Some of the pre-identified user groups (Table 1) were combined if enrolment was low, for reasons of confidentiality; the family caregiver group was combined with the older adults and family or friends group and the researchers group was combined with the implementation scientists group. No participants self-identified as data scientists or ethicists.

^b^ Including nonprofit and volunteer groups. 

^c^ Including students and fellows. 

^d^ No additional information available.

In Round 1, 19 (26%) participants suggested 44 new potential outcomes, rating 31 as critical. The participants suggested between 1 and 12 new items each (mean = 2). Only one of the 19 participants suggested more than four items.

Most of the suggestions were descriptive variables and not outcomes. For example, suggested community-level factors included “accessibility”; “community-level outcomes (beyond individual and caregiver)”; “neighbourhood area; power/resource shift to community (funding, paid roles, decision-making, etc.)”; and “social determinants of health.” Other suggested items related to person-level outcomes were already in the taxonomy (e.g. belonging, mental well-being, etc.). 

Seven items, proposed by four participants, related to equity: “access to health care; health outcomes; access to social determinants of health”; “cultural safety and inclusion”; “participatory governance; patient/participant voice; community voice”; “improved access to social determinants of health (housing, income, employment, social participation, etc.)”; “language services”; “that health equity be more of a global-level measurement than an individual level”; and “standardization of service applies to all categories.”

Only one of the items proposed in Round 1 was added to the survey in Round 2: “community benefits (increase in availability/stability/access for community services/resources, physical space improvements, social cohesion, etc.).” The team members involved in the adjudication process [MCA, AC] decided that this outcome was not a descriptor and that it could be related to social prescribing interventions that aim to increase benefits for the community.

In Round 2, 52 participants from eight user groups completed the survey (70% retention) (Table 2. The median rating values between groups and rounds were overall consistent. For user groups with five or more participants, the median (interquartile range [IQR]) rating values for Round 1 were as follows: community groups, 7 (2); health care providers, 7 (2); older adults and family or friends/caregivers, 7 (3); researchers and implementation scientists, 7 (3); and trainees, 7 (3). For Round 2, the median (IQR) values were as follows: community groups, 7 (2); health care providers, 6 (2); older adults and family or friends/caregivers, 7 (3); researchers and implementation scientists, 7 (4); and trainees,7(3).

None of the outcomes met the threshold for exclusion in either round of the Delphi survey and none were deleted. In Round 1, the mean percentage of people who rated an outcome as not important was 5.6% (standard deviation [SD]: 5.8%; range: 0–16.2%). In Round 2, the mean (SD) was 4.8% (SD: 6%; range: 0–21.2%). The “musculoskeletal and connective tissue” outcome was the only one considered “not important” (i.e. assigned a value of 1–3 points) by 20% of participants.

In December 2024, six team members [MCA, AC, ML, TP, KR, ST] met to discuss the findings, but no changes were made to the list of outcomes.

The list of outcomes, in order of the proportion of participants who rated them as critical in Round 2 of the Delphi survey, is shown in Table 3.

**Table 3:** Proportion of participants who rated outcomes “critical”a in Round 2 of the Delphi survey

Outcome	Proportion of participants who rated the outcome “critical,” %	
	Round 2 (n = 52)	Round 1 (n = 74)
Physiological/clinical – mental health (anxiety, depression, mood, etc.)	94	89
Life impact – physical functioning (frailty, physical activity, life activities)	92	84
Life impact – social functioning (belonging, friendship, social participation)	90	88
Life impact – emotional functioning/well-being (well-being, life satisfaction, loneliness, self-esteem, self-efficacy)	90	89
Physiological/clinical – general outcomes (disease burden, pain, number of chronic conditions)	82	77
Life impact – global QoL	80	74
Delivery of care – patient/carer satisfaction (person or family satisfaction with program, perceived benefits, expectations)	75	64
Resource use – economic (GP visits/calls/ health resource utilization/hospitalizations)	71	65
Life impact – personal circumstances (available resources [personal or community], needs)	67	66
Delivery of care – process, implementation and service outcomes (program acceptability, adoption, reach, maintenance, referral sources)	61	63
Resource use – need for further intervention (additional referrals based on identified needs, e.g. physiotherapy, occupational therapy, community programs, medications)	59	58
Delivery of care – adherence/compliance (adherence or completion of program)	59	50
Life impact – perceived health status	57	51
Adverse events (injuries or negative consequences resulting from engagement in programs)	55	54
Community benefits (increase in availability/stability/access for community services/resources, physical space improvements, social cohesion, etc.)^b^	54	Not applicable
Resource use – societal/carer burden (social support)	47	52
Mortality (all-cause and specific-cause survival/mortality and related outcomes)	39	36
Resource use – hospital visits	37	42
Life impact – cognitive functioning (cognition)	35	55
Life impact – role functioning (work)	24	26
Physiological/clinical – musculoskeletal and connective tissue (bone health, muscle strength)	21	27
Physiological/clinical – metabolism and nutrition (BMI, energy expenditure)	18	24

**Abbreviations: **BMI, body mass index; GP, general practitioner; QoL, quality of life. 

^a ^ Predefined as ≥ 75% of Delphi survey participants rating the outcome as critical (7–9 points on a 9-point scale from 1 for “not important” to 9 for “critical”) with < 20% of participants rating the item as not important (1–3 points). 

^b^ Suggested in Round 1 and added to the survey in Round 2. 

The outcomes that were rated as critical in both rounds of the Delphi survey by the older adults and family or friends/caregivers user group and by remaining user groups combined are shown in Table 4.

**Table 4:** Outcomes rated criticala in Rounds 2 and 1 of the Delphi survey, by all participants and by subgroups

Round 2: all participants (n = 52)	Round 2: remaining user groups combined^b^(n = 38)	Round 2: older adults + family/friends/caregivers user group (n = 14)	Round 1: all participants (n = 74)	Round 1: remaining groups participants^b^ (n = 50)	Round 1: older adults + family/friends group (n = 24)
Physiological/clinical – mental health Life impact – physical functioning Life impact – emotional functioning/well-being Life impact – social functioning Physiological/clinical – general outcomes Life impact – global QoL Delivery of care – patient/carer satisfaction	Life impact – social functioning Physiological/clinical – mental health Life impact – emotional functioning/well-being Life impact – physical functioning Life impact – global QoL Life impact – personal circumstances^c^ Physiological/clinical – general outcomes	Life impact – physical functioning Physiological/clinical – general outcomes Delivery of care – adherence/compliance^c^ Resource use – GP visits/hospitalizations^c^ Resource use – need for further intervention^c^ Physiological/clinical – mental health Adverse events^c^ Delivery of care – patient/carer satisfaction Life impact – emotional functioning/well-being	Life impact – emotional functioning/well-being Physiological/clinical – mental health Life impact – social functioning Life impact – physical functioning Physiological/clinical – general outcomes	Life impact – emotional functioning/well-being Physiological/clinical – mental health Life impact – social functioning Life impact – physical functioning Life impact – global QoL^c^	Physiological/clinical – general outcomes Life impact – physical functioning Physiological/clinical – mental health Life impact – cognitive functioning^c^ Life impact – emotional functioning/well-being Resource use – need for further intervention^c^ Life impact – social functioning Resource use – GP visits/hospitalizations ^c^

**Abbreviations: **GP, general practitioner; QoL, quality of life. 

^a^ Predefined as ≥ 75% of Delphi survey participants rating the outcome as critical (7–9 points on a 9-point scale from 1 for “not important” to 9 for “critical”) with < 20% of participants rating the item as not important (1–3 points). 

^b^ Participants who self-selected from one of the following user groups: community groups or social service providers; health care providers; link workers; policy-makers, managers, decisionmakers; researchers and implementation scientists; trainees (including students and fellows); other (no additional information available). 

^c^ Consensus on these outcomes was only reached in these subgroups. 

An overview of the operational and construct definitions for the final seven critical outcomes identified via the Delphi approach is shown in Table 5. 

**Table 5:** Definitions for outcomes rated criticala by all user groups in Round 2 of the Delphi survey

Outcome	Operational definition in the survey	Explanation of construct
Emotional functioning/well-being	Well-being, life satisfaction, loneliness, self-esteem, self-efficacy	“…an umbrella term for psychological concepts such as life satisfaction, life purpose and positive emotions, all of which are shown to be associated with decreased mortality and improved physical and mental functioning.”^34,p.136^
Global QoL	QoL	“… individuals’ perceptions of their position in life in the context of the culture and value systems in which they live and in relation to their goals, expectations, standards and concerns.”^35,p.551^
Mental health	Anxiety, depression, mood, etc.	“A state of mental well-being that enables people to cope with the stresses of life, to realize their abilities, to learn well and work well, and to contribute to their communities.”^36,p.8^
Social functioning	Belonging, friendship, social participation	“Social functioning is defined as how a person operates in their unique social environment (i.e. engagement in activities, connectedness with others and contributions to social roles).”^37,p.1989^
General outcomes	Disease burden, pain, number of chronic conditions	“Disease burden is an important indicator of the state of health of a population. It can be measured as the frequency (e.g. incidence and prevalence) of a condition or its effects including fatal and nonfatal health loss from disease (e.g. disability-adjusted life years) as well as the financial costs (e.g. direct health care costs and indirect health care expenditures related to lost income because of premature death).”^38,p.2031^
Physical functioning	Frailty, physical activity, life activities	“Physical functioning is recognized as the ability of an individual to carry out activities that require physical capability; these may range from basic self-care to more intense activities.”^39,p.2757^
Patient/carer satisfaction	Person or family satisfaction with program, perceived benefits, expectations	Identifying a universally accepted definition of patient satisfaction is problematic,^40^ as is deciding which elements (e.g. providers, health care system, etc.) to include.^41^ Patient satisfaction is thought to be a measure of people’s perceptions and expectations of care, processes and environment,^42^ and it is distinct from patient experience.^43^

**Abbreviation:** QoL, quality of life. 

^a^ Predefined as ≥ 75% of Delphi survey participants rating the outcome as critical (7–9 points on a 9-point scale from 1 for “not important” to 9 for “critical”) with < 20% of participants rating the item as not important (1–3 points). 

## Discussion

Consistent and reliable outcome measures are paramount in research and practice,^22^ especially in an emerging field like social prescribing. We used a Delphi approach to support future research trials in social prescribing^22^ by determining seven critical outcomes alongside important contextual factors. Although this study was relatively small and focused on Canada, we conducted the research according to published guidelines^44^-^46^ and included people from many different user groups. 

Many of the critical outcomes were psychosocial factors—social (elements and/or processes) and psychological (perceptions and meanings)^47^—such as well-being. Participants also proposed outcomes beyond the person-level, a signal to consider the context of the applied nature of this model of care. Specifically, in addition to identifying critical outcomes for future social prescribing research trials,^8^,^22^ this work acknowledges the environmental and personal contexts within which an intervention is tested given that social prescribing opportunities depend on contextual factors like availability, affordability, accessibility and others. 

This work complements the definition of social prescribing (also developed using a Delphi approach) that looks beyond person-level factors.^3^ This initial list of critical outcomes for evaluating social prescribing research trials and interventions’ should be regularly updated within a comprehensive evaluation framework. This is especially salient as social prescribing continues to evolve.

***Outcomes that matter most***

Older participants were well-represented in this study, an important consideration when developing a core outcome set,^13^ even though most participants lacked personal experience with social prescribing. In the subgroup analyses, the selections made by the older adults and family members or friends user group differed from those of the remaining user groups combined. Notably, older adults and their family members or friends rated cognition as a critical outcome in Round 1. This did not receive the same emphasis from the other six groups collectively; nor did it reach the 75% threshold in Round 2 (in which fewer older adults participated). Previous research suggests that older people may worry about cognitive loss,^48^,^49^ which may account for these findings in the subgroup analysis in Round 1.

Other distinct outcomes for the older adults and family or friends/caregivers user group relates to implementation factors; these were rated lower by the other user groups combined. Specifically, this user group rated the outcomes “adherence/compliance,” “resources needed for further intervention” and “adverse events” for inclusion. One plausible explanation for this disparity may be that the older adults took a broader perspective to evaluate social prescribing (based on their lived experience), while the remaining user groups focused on outcomes relevant to research trials. Alternatively, this user group may have found it challenging to engage in the Delphi study (as suggested in other work^50^) and the research process^51^ and may therefore have provided different rating scores.^52^ However, examination of the median values of the user group scores do not align with this observation. Further, a systematic review noted that core outcome sets (for routine care) that did not include patients and similar users were less likely to include outcomes from a “life impact” domain.^53^

Finally, “personal circumstances” (e.g. available resources [personal or community], needs)” exceeded the threshold for a critical outcome for participants from the remaining user groups. Although outcomes in this domain may not always be considered primary, they may contain key contextual data that help to situate findings within the bigger picture and address equity-related factors. This domain shares similar features to the community-level items proposed by participants in Round 1. Thus, although we identified critical outcomes that “matter,” these serve as a reminder to collect and consider other factors, especially those that promote equity within social prescribing. Collectively, participant ratings in this study provide insights into what may be important from their perspective and are valuable for an overall evaluation plan.

***Equity factors***

There is a need for routinely collected data on the social determinants of health,^54^ particularly as they relate to health equity.^55^ Recent research on social prescribing in Canada highlights the need to take health equity into account in future work,^56^ as part of grounding social prescribing within the Quintuple Aim.^57^ There are also examples of recent Canadian social prescribing initiatives that focused on health equity.^25^,^58^,^59^ There are numerous definitions for health equity, with many encompassing “the aim of achieving the highest level of health for all people, providing the opportunity to do so, and ensuring the absence of disparities.”^60^^,p.572^ However, information related to equity is not always consistently collected or considered within social prescribing.^61^ Specifically, evidence published to date has been more general and not disaggregated by personal characteristics,^61^ and equity-related factors are often missing in publications.^62^

Guiding literature and innovations are available to support identifying and collecting personal factors that describe study participants and their environments with the aim of improving equity or minimizing inequities. Examples include PROGRESS-Plus,^63^,^64^ the Screening for Poverty and Related Social Determinants to Improve Knowledge of and Links to Resources (SPARK) tool^54^ and the Diversity Minimal Item Set (DiMIS).^65^ These innovations, if adopted within research, may be a way to improve the inclusion and collection of data to inform the evaluation of social prescribing.

***The big picture***

The aim of our work was to identify outcomes considered critical to measure in social prescribing research based on an established Delphi process.^22^ No outcomes were excluded (i.e. deemed unimportant), and other descriptive factors were also proposed, particularly ones related to equity. These outcomes should also be incorporated within a larger evaluation framework. 

Our findings share similarities with existing frameworks, such as the International Classification of Functioning, Disability and Health, which situates health and disability within a larger context to account for environmental and personal factors.^66^ Still, social prescribing is not a single complex intervention^67^ but a series of relationships and interventions^67^,^68^ that take into account factors beyond person-level outcomes, for example, context. In particular, social prescribing aims to tackle health inequities,^69^ which may underpin the need for social prescribing in the first place.^9^ Health inequities are the “differences in health status or in the distribution of health resources between different population groups, arising from the social conditions in which people are born, grow, live, work and age.”^70^^,p.1^

In its current form, social prescribing is more focused on person-level outcomes (aligned with the critical outcomes identified in this study) and interventions. Several studies have described the challenges of using person-level interventions to address health disparities.^69^,^71^,^72^ Therefore, as social prescribing evolves, there is a need to regularly reconsider the evolving (and growing) social prescribing movement and, in particular, the practice and the people who use its services. There is also a need to articulate a conceptual understanding of social prescribing, including defining its potential mechanism(s) of action and core functions. 

Integrating the principles of the Quintuple Aim^57^ and the Ottawa Charter^56^ with those of social prescribing should also be considered. The focus of the Quintuple Aim, initially introduced by the Institute for Healthcare Improvement in 2007 as the Triple Aim, was on enhancing patient experiences and outcomes while reducing costs; it was later updated to include clinician well-being and health equity.^73^ The Ottawa Charter has five action items for health: public policies, supportive environments, community action, personal skills and health system changes.^74^ Together, these guiding frameworks focus on both the person and the wider community or society through understanding and addressing social determinants of health, for example.^56^ They aim to support people and address the environments within “which people are born, grow, live, work and age.”^70^^,p.1^ Thus, although this present study is a starting place for identifying a core outcome set, larger evaluation frameworks for social prescribing should be considered. Some are in development (for example, Elliott et al.,^75^ Caldern-Larraaga et al.^76^), while others already exist (for example, NHS England’s *Social Prescribing and Community-based Support: Summary Guide*^77^).

***Strengths and limitations***

We identified seven critical outcomes to consider in evaluations of social prescribing research and interventions and to understand, at least in part, what may be important to recipients such as the older adults and caregivers who participated in this study. 

However, the systematic review and Delphi processes have inherent limitations. For example, the 21 outcomes rated in this Delphi study were extracted from previous research studies by our 2024 evidence review.^9^ This list may reflect the early adoption and implementation of social prescribing interventions. As the social prescribing movement continues to grow, the core functions of social prescribing may evolve and other outcomes may be considered critical should the consensus process be repeated.

In addition, as we did not ask participants to provide detailed sociodemographic data, we do not know how representative our sample was of the overall population of Canadian adults in this age group (≥ 60 years). We also recognize most participants did not have specific experience delivering or receiving social prescribing or a Canadian perspective; together, these factors may limit the generalizability of the work.

Social prescribing is not a single complex intervention,^67^ but a collection of multiple complex interventions.^67^,^68^ Consequently, it may be challenging to identify critical outcomes that are relevant to all aspects of social prescribing, that go beyond person-level outcomes, and that include community-level outcomes (e.g. access to resources) and research-related outcomes (e.g. adverse events). Nonetheless, our findings may be beneficial for researchers and practitioners planning social prescribing research trials, which was the primary objective of our study. Consistency in the use of outcome measures may support evaluating the effectiveness of social prescribing interventions, enhance the synthesis of data in future evidence reviews and possibly support the practice of social prescribing.

We also recognize that social prescribing is growing and evolving, and that there may be differences in what matters to people receiving and those delivering social prescribing. This is not a new phenomenon,^13^,^78^ and everyone involved has a duty to continue exploring how to best measure and evaluate what is important to a wider group of interested parties.

***Next steps***

This and our previous study^9^ should be viewed as the start of developing acceptable and meaningful outcomes and evaluations for and by people affected by and who can affect social prescribing. In order to do this at a systems level, it is important to first develop a list of the core functions in social prescribing, similar to the four core primary care functions (the “4Cs of PC”) proposed for public health^79^ that address “better quality services, lower costs, less inequality in health care and better population health.”^79^^,p.1^ As such, our work should be seen as a “living document” that is regularly updated in different contexts over time. In addition, building on our umbrella review,^9^ more work is needed to define specific instruments (e.g. questionnaires, performance-based tests) to assess each outcome. 

We will release findings (via emails, presentations, surveys, websites, etc.) to receive feedback and external validation as part of our preplanned knowledge mobilization strategies.^6^

## Conclusion

Here we provide an initial list of critical and important outcomes to consider for social prescribing research; participants further provided feedback on environmental and personal (contextual) information, which should be considered within evaluation frameworks (the bigger picture). This first iteration of a core outcome set for social prescribing will require regular updates, as results are likely to vary as a result of numerous factors, including the ongoing evolution of social prescribing.

## Acknowledgements

We thank all the study participants for their contributions to this work. 

MCA and TP gratefully acknowledge the support of the Canada Research Chairs Program. AMC acknowledges the support of the Canadian Institutes of Health Research through the Patient-Oriented Research Awards – Transition to Leadership Stream, Phase 2 Award (reference number 188352). ST acknowledges the support of the Research Trainee Award from Michael Smith Health Research BC.

## Funding

Social Sciences and Humanities Research Council of Canada and the UBC Health Innovation Funding Investment (HIFI) Awards.

## Conflicts of interest

None.

## Authors’ contributions and statement

MCA: Conceptualization, methodology, analysis, supervision, visualization, writing—original draft, writing—review and editing.

AMC, KM: Conceptualization, methodology, analysis, writing—review and editing.

ML, TI, GSN, WBM, TP, RP, KLR, BS, ST: writing—review and editing.

All authors agreed to the published version of the manuscript.

The content and views expressed in this article are those of the authors and do not necessarily reflect those of the Government of Canada.

## Data availability statement

Data cannot be shared publicly because we did not ask permission from participants.

## References

[B01] Morse DF, Sandhu S, Mulligan K, Tierney S, Polley M, Giurca B, et al (2022). Global developments in social prescribing. BMJ Glob Health.

[B02] Khan H, Giurca BC, Burgess RA, Genn H, Dixon M, Leitch A, et al Social prescribing around the world: a world map of global developments in social prescribing across different health system contexts: 2024. Global Social Prescribing Alliance.

[B03] Muhl C, Mulligan K, Bayoumi I, Ashcroft R, Godfrey C (2023). Establishing internationally accepted conceptual and operational definitions of social prescribing through expert consensus: a Delphi study. BMJ Open.

[B04] Percival A, Newton C, Mulligan K, Petrella RJ, Ashe MC (2022). Systematic review of social prescribing and older adults: where to from here. Fam Med Community Health.

[B05] Husk K, Elston J, Gradinger F, Callaghan L, Asthana S (2019). Social prescribing: where is the evidence. Br J Gen Pract.

[B06] Esfandiari E, Chudyk AM, Grover S, Lau EY, Hoppmann C, Mortenson WB, et al (2023). Social Prescribing Outcomes for Trials (SPOT): protocol for a modified Delphi study on core outcomes. PLoS One.

[B07] Flynn S, Sundaresan S, Caffrey L (2024). Putting outcomes into practice: the implementation of a framework of outcome measures within a child and family service. Br J Soc Work.

[B08] Williamson PR, Altman DG, Bagley H, Barnes KL, Blazeby JM, Brookes ST, et al (2017). The COMET handbook: version 1.0. Williamson PR, Altman DG, Bagley H, Barnes KL, Blazeby JM, Brookes ST, et al.

[B09] Ashe MC, Santos IK, Alfares H, Chudyk AM, Esfandiari E (2024). Outcomes and instruments used in social prescribing: a modified umbrella review. Health Promot Chronic Dis Prev Can.

[B10] Population projections for Canada, provinces and territories, 2021 to 2068, 2022 [Internet]. Statistics Canada.

[B11] Grover S, Sandhu P, Nijjar GS, Percival A, Chudyk AM, Liang J, et al (2023). Older adults and social prescribing experience, outcomes, and processes: a meta-aggregation systematic review. Public Health.

[B12] Ooi LL, Liu L, Roberts KC, py G, Capaldi CA (2023). Social isolation, loneliness and positive mental health among older adults in Canada during the COVID-19 pandemic. Health Promot Chronic Dis Prev Can.

[B13] Biggane AM, Brading L, Ravaud P, Young B, Williamson PR (2018). Survey indicated that core outcome set development is increasingly including patients, being conducted internationally and using Delphi surveys. Trials.

[B14] Chudyk AM, Horrill T, Waldman C, Demczuk L, Shimmin C, Stoddard R, et al (2022). Scoping review of models and frameworks of patient engagement in health services research. BMJ Open.

[B15] Dalkey N, Helmer O (1963). An experimental application of the Delphi method to the use of experts. Manage Sci.

[B16] Dalkey N (1969). An experimental study of group opinion: the Delphi method. Futures.

[B17] Cantrill J, Sibbald B, Buetow S (1996). The Delphi and nominal group techniques in health services research. Int J Pharm Pract.

[B18] Gustafson DH, Shukla RK, Delbecq A, Walster GW (1973). A comparative study of differences in subjective likelihood estimates made by individuals, interacting groups, Delphi groups, and nominal groups. Organ Behav Hum Perform.

[B19] Graefe A, Armstrong JS (2011). Comparing face-to-face meetings, nominal groups, Delphi and prediction markets on an estimation task. Int J Forecast.

[B20] Veugelers R, Gaakeer MI, Patka P, Huijsman R (2020). Improving design choices in Delphi studies in medicine: the case of an exemplary physician multi-round panel study with 100% response. BMC Med Res Methodol.

[B21] Prinsen CA, Vohra S, Rose MR, Jones S, Ishaque S, Bhaloo Z, et al (2014). Core Outcome Measures in Effectiveness Trials (COMET) initiative: protocol for an international Delphi study to achieve consensus on how to select outcome measurement instruments for outcomes included in a ‘core outcome set’. Trials.

[B22] Williamson PR, Altman DG, Blazeby JM, Clarke M, Devane D, Gargon E, et al (2012). Developing core outcome sets for clinical trials: issues to consider. Trials.

[B23] Dodd S, Clarke M, Becker L, Mavergames C, Fish R, Williamson PR (2018). A taxonomy has been developed for outcomes in medical research to help improve knowledge discovery. J Clin Epidemiol.

[B24] Freeman RE (1984). Strategic management: a stakeholder approach. Pitman.

[B25] Bhatti S, Rayner J, Pinto AD, Mulligan K, Cole DC (2021). Using self-determination theory to understand the social prescribing process: a qualitative study. BJGP Open.

[B26] Lin MC, Park G, Ashe MC (2024). Integrating social prescribing in a Canadian regional health system to support healthy aging. Health Promot Chronic Dis Prev Can.

[B27] Nasa P, Jain R, Juneja D (2021). Delphi methodology in healthcare research: how to decide its appropriateness. World J Methodol.

[B28] Dougherty M, Tompkins T, Zibrowski E, Cram J, Ashe MC, Bhaskar LT, et al (2024). Coproduction in social prescribing initiatives: protocol for a scoping review. JMIR Res Protoc.

[B29] Thomas G, Lynch M, Spencer LH (2021). A systematic review to examine the evidence in developing social prescribing interventions that apply a co-productive, co-designed approach to improve well-being outcomes in a community setting. Int J Environ Res Public Health.

[B30] Plamondon K, Ndumbe-Eyoh S, Shahram S, Graham ID, Rycroft-Malone J, Kothari A, McCutcheon C (2022). 2.2 Equity, power, and transformative research coproduction. Wiley.

[B31] Dabbs C (2024). Social prescribing: community power and the community paradigm. Clinics Integr Care.

[B32] Schiller C, Winters M, Hanson HM, Ashe MC (2013). A framework for stakeholder identification in concept mapping and health research: a novel process and its application to older adult mobility and the built environment. BMC Public Health.

[B33] Grande C, Kaczorowski J (2023). Rating versus ranking in a Delphi survey: a randomized controlled trial. Trials.

[B34] Feller SC, Castillo EG, Greenberg JM, Abascal P, Horn R (2018). Emotional well-being and public health: proposal for a model national initiative. Public Health Rep.

[B35] Harper A, Power M (1998). Development of the World Health Organization WHOQOL-BREF quality of life assessment. Psychol Med.

[B36] Lewis S, Freeman M, Ommeren M, Chisholm D, Siegl OG, Kestel D World mental health report: transforming mental health for all. World Health Organization.

[B37] Madrigal C, Bower E, Simons K, Gillespie SM, Orden K, Mills WL (2021). Assessing social functioning during COVID-19 and beyond: tools and considerations for nursing home staff. J Am Med Dir Assoc.

[B38] Udompap P, Kim D, Kim WR (2015). Current and future burden of chronic nonmalignant liver disease. Clin Gastroenterol Hepatol.

[B39] Palmer E, Johar I, Little DJ, Karlsson N (2024). Development of a conceptual model of physical functioning limitations experienced by patients with late-stage chronic kidney disease: a qualitative interview study. Adv Ther.

[B40] Crow H, Gage H, Hampson S, Hart J, Kimber A, Storey L, et al (2003). The measurement of satisfaction with health care: implications for practice from a systematic review of the literature. Health Technol Assess.

[B41] Bleich SN, Ozaltin E, Murray CJ (2009). How does satisfaction with the health-care system relate to patient experience. Bull World Health Organ.

[B42] Lleshi S, Mustafa B, Sci J (2025). Patient satisfaction with nursing care and information received from nurses. Multidiscipl Sci J.

[B43] Bull C (2021). Patient satisfaction and patient experience are not interchangeable concepts. Int J Qual Health Care.

[B44] nger S, Payne SA, Brine J, Radbruch L, Brearley SG (2017). Guidance on Conducting and REporting DElphi Studies (CREDES) in palliative care: recommendations based on a methodological systematic review. Palliat Med.

[B45] Gattrell WT, Logullo P, Zuuren EJ, Price A, Hughes EL, Blazey P, et al (2024). ACCORD (ACcurate COnsensus Reporting Document): a reporting guideline for consensus methods in biomedicine developed via a modified Delphi. PLoS Med.

[B46] Niederberger M, Schifano J, Deckert S, Hirt J, Homberg A, berich S, et al (2024). Delphi studies in social and health sciences – recommendations for an interdisciplinary standardized reporting (DELPHISTAR). PLoS One.

[B47] Stansfeld S, Rasul F, Steptoe A (2006). Psychosocial factors, depression and illness. Cambridge University Press.

[B48] Werner P, AboJabel H, Maxfield M (2021). Conceptualization, measurement and correlates of dementia worry: a scoping review. Arch Gerontol Geriatr.

[B49] Niechcial MA, Vaportzis E, Gow AJ (2019). People’s views on preserving thinking skills in old age. Educ Gerontol.

[B50] Barrington H, Young B, Williamson PR (2021). Patient participation in Delphi surveys to develop core outcome sets: systematic review. BMJ Open.

[B51] Biggane AM, Williamson PR, Ravaud P, Young B (2019). Participating in core outcome set development via Delphi surveys: qualitative interviews provide pointers to inform guidance. BMJ Open.

[B52] Gargon E, Gurung B, Medley N, Altman DG, Blazeby JM, Clarke M, et al (2014). Choosing important health outcomes for comparative effectiveness research: a systematic review. PLoS One.

[B53] Kearney A, Williamson PR, Dodd S (2024). A review of core outcome sets (COS) developed for different settings finds there is a subset of outcomes relevant for both research and routine care. J Clin Epidemiol.

[B54] Adekoya I, Delahunty-Pike A, Howse D, Kosowan L, Seshie Z, Abaga E, et al (2023). Screening for poverty and related social determinants to improve knowledge of and links to resources (SPARK): development and cognitive testing of a tool for primary care. BMC Prim Care.

[B55] Mary K, Wakefield MK, Williams DR, Menestrel S, Flaubert JL (2021). Social determinants of health and health equity. National Academies Press.

[B56] Mulligan K, Card KG, Allison S (2024). Social prescribing in Canada: linking the Ottawa Charter for Health Promotion with health care’s Quintuple Aim for a collaborative approach to health. Health Promot Chronic Dis Prev Can.

[B57] Nundy S, Cooper LA, Mate KS (2022). The Quintuple Aim for health care improvement: a new imperative to advance health equity. JAMA.

[B58] Ramirez S, Beaudin N, Rayner J, Price N, Townsend D (2024). Black-focused social prescribing: the importance of an Afrocentric approach. Health Promot Chronic Dis Prev Can.

[B59] Kadowaki L, Symes B, Lalji K, Park G, Giannasi W, Hystad J, et al (2024). Building the capacity of older adults and community: findings from a developmental evaluation of United Way British Columbia's social prescribing programs for older adults. Health Promot Chronic Dis Prev Can.

[B60] Hoyer D, Dee E, O’Leary MS, Heffernan M, Gelfand K, Kappel R, et al (2022). How do we define and measure health equity. J Public Health Manag Pract.

[B61] Khan K, Tierney S, Owen G (2024). Applying an equity lens to social prescribing. Applying an equity lens to social prescribing. J Public Health (Oxf).

[B62] Martinez RA, Smith NR, Wilbur RE, Andrabi N, Goodwin AN, Zivich PN Threats to equity: missing methodological details about race and ethnicity in health research. Health Affairs Forefront.

[B63] PROGRESS-Plus [Internet]. Cochrane.

[B64] O’Neill J, Tabish H, Welch V, Petticrew M, Pottie K, Clarke M, et al (2014). Applying an equity lens to interventions: using PROGRESS ensures consideration of socially stratifying factors to illuminate inequities in health. J Clin Epidemiol.

[B65] Stadler G, Chesaniuk M, Haering S, Roseman J, burger VM, Martina S, et al (2023). Diversified innovations in the health sciences: proposal for a Diversity Minimal Item Set (DiMIS). Sustain Chem Pharm.

[B66] Leonardi M, Lee H, Kostanjsek N, Fornari A, Raggi A, Martinuzzi A, et al (2022). 20 Years of ICF—International Classification of Functioning, Disability and Health: uses and applications around the world. Int J Environ Res Public Health.

[B67] Husk K, Blockley K, Lovell R, Bethel A, Lang I, Byng R, et al (2020). What approaches to social prescribing work, for whom, and in what circumstances. Health Soc Care Community.

[B68] Hazeldine E, Gowan G, Wigglesworth R, Pollard J, Asthana S, Husk K (2021). Link worker perspectives of early implementation of social prescribing: a ‘researcher-in-residence’ study. Health Soc Care Community.

[B69] Gibson K, Pollard TM, Moffatt S (2021). Social prescribing and classed inequality: a journey of upward health mobility. Soc Sci Med.

[B70] Health inequities and their causes [Internet]. WHO.

[B71] Mackenzie M, Skivington K, Fergie G (2020). “The state they’re in”: unpicking fantasy paradigms of health improvement interventions as tools for addressing health inequalities. Soc Sci Med.

[B72] Moscrop A (2023). Social prescribing is no remedy for health inequalities. BMJ.

[B73] Itchhaporia D (2021). The evolution of the Quintuple Aim: health equity, health outcomes, and the economy. J Am Coll Cardiol.

[B74] Wilberg A, Saboga-Nunes L, Stock C (2021). Are we there yet. J Public Health (Berl.).

[B75] Elliott M, Davies M, Davies J, Wallace C (2022). Exploring how and why social prescribing evaluations work: a realist review. BMJ Open.

[B76] aga S, Milner Y, Clinch M, Greenhalgh T, Finer S (2021). Tensions and opportunities in social prescribing. BJGP Open.

[B77] Personalised care: social prescribing and community-based support—summary guide [Internet]. NHS England and NHS Improvement.

[B78] Oravec N, Arora RC, Bjorklund B, Gregora A, Monnin C, Dave MG, et al (2023). Patient and caregiver preferences and prioritized outcomes for cardiac surgery: a scoping review and consultation workshop. J Thorac Cardiovasc Surg.

[B79] Jimenez G, Matchar D, Koh GC, Tyagi S, Kleij RM, Chavannes NH, et al (2021). Revisiting the four core functions (4Cs) of primary care: operational definitions and complexities. Prim Health Care Res Dev.

